# Adaptation and Validation of the Bahasa Version of the Diabetes Obstacles Questionnaire-Short Version Among Indonesians With Type 2 Diabetes Mellitus

**DOI:** 10.1097/jnr.0000000000000704

**Published:** 2025-09-15

**Authors:** Siti FADLILAH, Wahyu Rochdiat MURDHIONO, Mohammad Hendra Setia LESMANA, Herry SUSANTO, Ratsiri THATO, Yohanes Andy RIAS, Hsiu Ting TSAI

**Affiliations:** 1 School of Nursing, College of Nursing, Taipei Medical University, Taipei, Taiwan, ROC; 2 Program Study of Nursing, Universitas Respati Yogyakarta, Yogyakarta, Indonesia; 3 Department of Mental Health and Community Nursing, Faculty of Medicine, Public Health, and Nursing, Universitas Gadjah Mada, Yogyakarta, Indonesia; 4 Faculty of Nursing, Universitas Islam Sultan Agung, Semarang, Indonesia; 5 Faculty of Nursing, Chulalongkorn University, Bangkok, Thailand; 6 Center of Excellence for Enhancing Well-being in Vulnerable and Chronic Illness Populations, Faculty of Nursing, Chulalongkorn University, Bangkok, Thailand; 7 Faculty of Health, College of Nursing, Institut Ilmu Kesehatan Bhakti Wiyata, Kediri, Indonesia; 8 Post-Baccalaureate Program in Nursing, College of Nursing, Taipei Medical University, Taipei, Taiwan, ROC

**Keywords:** diabetes mellitus, psychometric, quality of life, reliability, validity

## Abstract

**Background:**

The Diabetes Obstacles Questionnaire-Short Version is a tool for measuring the quality of life in patients with diabetes mellitus. However, the validity and reliability of this tool has never been tested in Indonesia.

**Purpose:**

This study was designed to adapt and validate a Bahasa Indonesia version of the Diabetes Obstacles Questionnaire-Short Version for Indonesians with type 2 diabetes mellitus.

**Methods:**

This was a cross-sectional study. Data were collected from July to November 2023, and accidental sampling was used to recruit and enroll 1,116 participants. Convergence testing used the Diabetes Quality of Life questionnaire. The validity test used construct and convergent validities with significant Pearson correlations, and construct validity included exploratory factor analysis and confirmatory factor analysis. Reliability testing used Cronbach’s alpha, composite reliability, and average variance extracted.

**Results:**

The results of the exploratory factor analysis showed nine factors with a factor loading per item of >.45, a Kaiser-Meyer-Olkin test score of .915, and significant Bartlett’s test of sphericity results. The Cronbach’s alpha value for all items was .930, and those of individual factors ranged from .730 to .848. The test-retest results with interclass correlation coefficients ranged from .910 to .973. The results of the confirmatory factor analysis indicate that this instrument has an acceptable model fit.

**Conclusions/Implications for Practice:**

The Bahasa Indonesia Diabetes Obstacles Questionnaire-Short Version produces valid and reliable results for measuring quality of life in people with type 2 diabetes mellitus in Indonesia. The sufficient but small number of items allows the questionnaire to be completed relatively quickly, making it useful in clinical settings. The subscales in this instrument may be used simultaneously or separately based on individual needs.

## Introduction

According to the World Health Organization, as many as 41 million people die every year from noncommunicable diseases, representing some 74% of the global death rate. Diabetes mellitus (DM) ranks fourth among leading noncommunicable diseases, and its prevalence and incidence have also significantly increased in recent decades (World Health Organization, 2023). China has the highest number of people with DM (PWDM) at 140.9 million, followed by India with 74.2 million, the United States with 32.2 million, and Indonesia with 19.5 million. Worldwide, PWDM are projected to reach 643 million in 2030 and 783 million in 2045 (International Diabetes Federation, 2021). DM, as a chronic disease, is an unpleasant and frightening experience for sufferers (Al-Qerem et al., 2022; Mansour et al., 2023).

Prevalent problems caused by DM relate to its associated complications, including high risks of nonvascular and vascular complications (microvascular and macrovascular complications; Aikaeli et al., 2022). DM is the leading cause of heart problems, stroke, kidney failure, leg amputations, and even blindness (Mansour et al., 2023). As a result, these complications result in a higher mortality rate for PWDM than nondiabetics (Tomic et al., 2022). PWDM report experiencing physical and mental problems as well as changes in lifestyle. Their condition necessitates their regularly taking medication and depending on insulin and the support of family, as well as exposes them to opposing views or even the rejection of others, leading to reduced quality of life (QoL; AbuAlhommos et al., 2022; Al-Qerem et al., 2022; Tamornpark et al., 2022).

QoL is defined as a person’s perception of their position in life compared with the goals, expectations, standards, and concerns reflected in their cultural and personal values. Health-related QoL (HRQoL), a subset of QoL, refers to QoL in the context of individual health only (Cai et al., 2021). HRQoL in PWDM includes physical and mental perceptions, community-level resources, and conditions, with mental perceptions including health conditions and socioeconomic status, and conditions including practices influencing health perceptions and functional status (Trikkalinou et al., 2017). QoL is one of the criteria used in predicting a person’s capacity to manage DM, playing a vital role in predicting treatment response, evaluating the impact of various management regimens, and determining further interventions through concurrent consideration of their biomedical and psychosocial aspects (Gupta et al., 2021). Health providers widely recognize good QoL as the ultimate goal of all interventions and as measurable. Thus, QoL is essential in determining the effectiveness and burden of treatment on PWDM (PrasannaKumar et al., 2018). In light of the above, a valid and reliable instrument is needed for the early detection of obstacles and problems in people with DM, as well as for early therapy and management to prevent severe conditions.

Many instruments have been developed to measure QoL and its factors of influence. The diversity of instruments currently used to measure HRQOL in PWDM was explained in a recent literature review (Oluchi et al., 2021), with the Diabetes Obstacles Questionnaire (DOQ) identified as an effective measure of QoL in type 2 DM (T2DM) patients. In 2000, to explore how to fill the gap regarding the concept of QoL in PWDM, researchers started the EUROBSTACLE project to identify the ethnicity, culture, and health system-related obstacles faced by PWDM. The 113 items identified were organized into eight discrete factors and scored using a 5-point Likert scale (Vermeire et al., 2007). The results of this project were then tested for validity and reliability, with the results of the original version of the DOQ-78 items shown to be valid and reliable. These items were then organized into eight factors and scored using a 5-point Likert scale (Hearnshaw et al., 2007), and the DOQ-78 was subsequently confirmed for validity and reliability in other studies (Pilv et al., 2012). One weakness of the DOQ-78 is its large number of items, making it time-consuming to complete and difficult for health service providers to manage. Thus, a short version of the DOQ, the DOQ-30, was developed and confirmed using validity and reliability analyses. The DOQ-30 consists of 30 items, scored using a 5-point Likert scale, organized into the following 9 factors: relationship with medical professionals, support from friends and family, knowledge about the disease, lifestyle changes, exercise, self-monitoring, uncertainty about consultation, treatment, and insulin use. The total score for the DOQ-30 ranges from 30 to 150, with higher scores indicating better QoL. The DOQ-30 has been tested for validity and reliability in six European countries, with exploratory factor analysis (EFA) used in construct validity testing (Pilv et al., 2016).

The DOQ has been translated into several other languages, including Dutch (Vandekerckhove et al., 2009) and Turkish (Kahraman et al., 2016), and subjected to a variety of validity and reliability test methods. All previously conducted studies used the DOQ-78 scale with 78 items. However, the DOQ-30 has never been translated into languages other than the original English or subjected to other validity and reliability test methods. The increasing need to collect valid and reliable data on the QoL of Indonesians with DM makes adapting and validating a DOQ-30 for use in Indonesia imperative. DOQ-30 is a valid instrument that comprehensively shows the obstacles experienced by people with T2DM and allows QoL to be assessed more comprehensively based on its physical, mental, social, and conditions aspects without requiring significant time to complete. Therefore, this study was designed to adapt and validate a Bahasa Indonesia version of the DOQ-30 for Indonesians with T2DM. In line with the original developer, we explored data fit using factor loadings and also analyzed the internal consistency and construct and convergent validities of the translated instrument. This study is expected to provide a valid and reliable instrument that may be used in the early detection of obstacles and problems in people with T2DM, allowing early therapy and management to be carried out to prevent the onset/exacerbation of more severe conditions.

## Methods

### Design, Participants, and Settings

This study was carried out from July to November 2023 using a cross-sectional design. Data were collected at public health centers in Yogyakarta, Bali, West Nusa Tenggara, East Nusa Tenggara, Central Java, East Java, East Kalimantan, North Kalimantan, West Kalimantan, Central Kalimantan, North Maluku, West Java, Jakarta, Lampung, and Banten provinces in Indonesia. All of the participants were patients with physician-diagnosed T2DM (confirmed via patient medical records). Based on a previous related study, the minimum sample size for studies on this topic is calculated with at least 10 participants per item (White, 2022). As the DOQ-30 comprises 30 items, the minimum sample size was calculated as 300. The 1,116 participants ultimately enrolled in this study were randomly assigned into either the exploratory factor analysis (EFA) group (558 participants) and or the confirmatory factor analysis (CFA) group (558 participants). The interclass correlation coefficient (ICC) was used for the test-retest reliability analysis. Based on previous studies, increasing confidence interval precision requires at least 100 subjects (Mokkink et al., 2023). Thus, 100 participants were enrolled to evaluate the ICC and completed the questionnaire 14 days after filling in the same questionnaire for the first time. We used an accidental sampling technique to obtain previously selected participants based on inclusion and exclusion criteria, with the inclusion criteria including being willing to participate, aged 17–74 years, able to communicate well, and able to read and write. The exclusion criterion was experiencing or having a history of mental disorders.

### Procedures

The original English version of the DOQ-30 was translated into Bahasa Indonesia after permission had been obtained from the original author (Pilv et al., 2016) via personal communication on June 12, 2023. For the psychometric test procedure, the guidelines proposed by Sousa and Rojjanasrirat were followed (Sousa & Rojjanasrirat, 2011). The two phases of this study are described below.

#### Phase 1: Translation and Content Validity Analysis

A committee approach with two bilingual translators and three expert reviewers was used for the translation and nine experts were involved in the content validity analysis.

##### 1. Forward Translation

The original English version of the DOQ-30 questionnaire was translated into Bahasa Indonesia by two independent translators working as nurses in, respectively, Taiwan and the United Kingdom, who were fluent in both Bahasa Indonesia and English.

##### 2. Comparison of the Translated Versions: Synthesis I

The two translations were compared by the researchers and discussed with professional team workers to prepare a new draft version I.

##### 3. Blind Backward Translation

The new draft version I was translated into English independently by a professional English teacher and an English-Indonesian translation institute.

##### 4. Comparison of the Back-Translated Versions: Synthesis II

The two backward translations were compared with the original instrument. Due to time and distance issues, team discussions with the translators were conducted online. The differences between the two backward translations were minor, resulting in no differences in either the meaning or content of the items, and were thus resolved by consensus. The results of these discussions became the new draft version II.

##### 5. Pilot Testing of the Pre-Final Version

Based on a recommended 10–40 participants for pilot testing, 20 individuals (10 T2DM and 10 non-DM subjects) participated in the pilot test. Beyond reducing costs and increasing efficiency, pilot studies involving non-DM subjects provide advantages such as improved generalizability, creating normative data sets, and providing essential data for identifying response range types (Teresi et al., 2022). Each participant was asked for their assessment of the clarity and ease of understanding of each item using a binary answer of “clear” or “unclear.” Content validity testing was conducted with a nine-person expert panel consisting of a doctor, one public health specialist, one psychiatric nurse working as an academic, one nurse at a mental hospital, one community nurse, two medical-surgical nurses working as academics, and two medical-surgical nurses at a hospital. The content validity index (CVI) calculation used Scale-CVI Average (S-CVI/ave). The first step was calculating the nine experts’ average proportion of relevant items. Then, the average Item-Level content validity index (I-CVI) was calculated as the total number of items rated as relevant by the nine experts divided by the total number of items (30).

##### 6. Preliminary Psychometric Testing With a Bilingual Sample

This step was not done in this study due to the limited number of participants able to communicate in both Bahasa Indonesia and English.

#### Phase 2: Psychometric Analysis

Construct validity (EFA and CFA) and convergent validity were used to assess the validity and internal consistency was used to assess the reliability of the DOQ-30.

##### 1. Psychometric Testing

Individuals with T2DM were recruited from public health centers for psychometric testing of the final Bahasa Indonesia version of the DOQ-30. The authors were assisted by 25 research assistants during the data collection process in 15 provinces. These assistants were all nurses who worked at the public health center in each research location. In the run-up to the study, the assistants were trained to explain the research details to equalize perceptions in the data collection. All PWDM who visited these public health centers for an examination and met the criteria were invited to be participants, with those willing to complete the study questionnaire.

### Measures

#### Demographic Characteristics

Demographic data were measured using a self-report questionnaire designed to gather information on age, gender, marital status, educational level, income, ethnicity, occupation, family medical history, health insurance status, and income.

#### DOQ-30

The short version of the DOQ, known as the DOQ-30, developed by Pilv et al. (2016), was used in this study. In the original study, loading factors for individual items ranged from .584 to .818, and Cronbach’s alpha values for individual factors ranged from .75 to .89 (Pilv et al., 2016). The scoring method used by previous researchers was followed in this study. The Bahasa Indonesian version of the DOQ-30 (DOQ-30-BI) consists of 30 items scored on a 5-point Likert scale with answer options of *never* (score 5), *almost never* (score 4), *sometimes* (score 3), *often* (score 2), and *always* (score 1). Total possible scale scores range from 30 to 150, with higher scores indicating better QoL.

#### Diabetes Quality of Life (DQoL) Questionnaire

Convergent validity was conducted to determine the extent of the relationship between the DOQ-30-BI and another similar instrument used to measure PWDM QoL, the Diabetes Quality of Life (DQoL) questionnaire. The DQoL questionnaire, developed by the Diabetes Control and Complications Trial Research Group (The DCCT Research Group, 1988) for the diabetes control and complications trial (DCCT) group, consists of 46 items in the four domains of satisfaction, impact, social and vocational issues, and worry about the effects of diabetes in the future. Questionnaire items are scored using a 5-point Likert scale (1–5), with higher total scores indicating better QoL (The DCCT Research Group, 1988). The Bahasa Indonesia version of the DQoL questionnaire had been previously created with the permission of the original authors (Farahdina, 2014). The construct validity test of the DQoL Bahasa Indonesia version shows data from the CFA with χ^2^/degrees of freedom (*df*) of 1.14, and a root mean squared error of approximation (RMSEA) of .03, indicating good model fit (Farahdina, 2014).

### Data Analysis

Data analysis was conducted on SPSS ver. 27.0 and SPSS AMOS ver. 26.0 (IBM Corp., Armonk, NY, USA). Data characteristics are presented as number, frequency distribution, mean, and *SD* for participant demographics and as minimum, maximum, mean, *SD*, skewness, and kurtosis values for each DOQ-30 item. Skewness and kurtosis were used to assess the shape of the data distribution, with acceptable skewness ranges of ±2 for normally distributed data and ±4 for the kurtosis coefficient (Mishra et al., 2019). The results indicate no data were missing.

In the EFA, principal axis factoring and direct oblimin rotation were used for extraction. Principal axis factoring is a least-squares estimate of model factors, and the given approach focuses on correlations, so it is preferred for causal modeling (Grieder & Steiner, 2022). Direct oblimin is an oblique (nonorthogonal) rotation method that minimizes cross-product loadings to simplify factors (Akhtar-Danesh, 2023). A fixed number of extracted factors (9), the same as the original version from the DOQ-30 developer, was used (Pilv et al., 2016). A factor loading of >.45 was used to identify each item reflecting the factor. The loading factor criteria were <.3 = weak, .3–.5 = moderate, and >.5 = good. For the EFA, data adequacy was confirmed using both the Kaiser-Meyer-Olkin (KMO) test and Bartlett’s test of sphericity, with a KMO value of >.6 and a significant Bartlett’s test of sphericity of *p*<.001 indicating data adequacy (Shrestha, 2021). The convergent validity was analyzed using the average variance extracted (AVE) and composite reliability (CR), with acceptable AVE and CR values defined as >.50 and >.7, respectively (Cheung et al., 2024). Pearson correlation statistics were used to measure the relationship between constructs, with adequate discriminant validity indicated when the square root of the AVE of each construct exceeds the correlation value of the other constructs (Cruchinho et al., 2024).

A CFA was performed to confirm data model fit and establish the quality for fitting the assessment model. Acceptable data model fit was defined in this study as a Chi-square test value (χ^2^)/*df* ≤3 (Kline, 2023), goodness-of-fit index (GFI) and adjusted GFI (AGFI) values of .80–.90 (Ximénez et al., 2022), an incremental fit index (IFI) value >.9 (Pehlivan et al., 2024), a Tucker-Lewis index (TLI) value ≥.80, a comparative fit index (CFI) value ≥.85 (Kline, 2023), and RMSEA and standardized root mean square residual (SRMR) values ≤.08 (Gegenfurtner, 2022).

Cronbach’s alpha was used to test the internal consistency reliability. A consistency reliability analysis was conducted on the 558 participants previously used for the EFA test, with a Cronbach’s alpha value ≥.70 indicating good internal consistency and a total item correlation of ≥.30 deemed to be adequate (Green et al., 2016). ICC was employed to analyze the test-retest reliability of the DOQ-30-BI, with ICC value criteria values of .75–.90 indicating good reliability results and values >.90 indicating perfect reliability (ten Hove et al., 2022).

### Ethical Considerations

This study was approved by both the Ethics Research Committee of Universitas Respati Yogyakarta (no. 090.3/FIKES/PL/V/2023) and the Taipei Medical University-Joint Institutional Board (no. N202308068).

## Results

### Participant Characteristics

Participant characteristics are summarized in Table 1. The 1,116 participants in this study had a mean (*SD*) age of 55.79 (9.90) years and fasting blood glucose (FBG) level of 203.57 (72.56) g/dL. The mean (*SD*) total score for all DQoL-30 items was 126.06 (16.11), and the total score for all DQoL items was 180.22 (22.48). Over half of the participants were female (53.3%), one-third (32.5%) had completed senior high school, 29.1% were working as private staff, 80.9% were ethnic Javanese, 58.5% had no family history of DM, 84.3% were married, 89.5% had national health insurance (NHI), and 58.2% earned <2,500,000 Indonesian Rupiah (IDR) per month.

**Table 1:** Participant Demographics (*N*=1,116)

Characteristic	Mean (*SD*)
Age (years)	55.79 (9.90)
FBG (mg/dL)	203.57 (72.56)
DOQ-30	126.06 (16.11)
DQoL	180.22 (22.48)
	*n* (%)
Gender	
Male	521 (46.7)
Female	595 (53.3)
Educational level	
None	34 (3.0)
Elementary school	305 (27.3)
Junior high school	163 (14.6)
Senior high school	363 (32.5)
Bachelor’s	228 (20.5)
Master’s	16 (1.4)
Doctor of Philosophy	7 (0.6)
Occupation	
None	175 (15.7)
Farmer	164 (14.7)
Housewife	146 (13.1)
Civil servant	77 (6.9)
Retired	28 (2.5)
Private staff	325 (29.1)
Others	201 (18.0)
Ethnicity	
Javanese	903 (80.9)
Non-Javanese	213 (19.1)
Family history of DM	
No	653 (58.5)
Yes	463 (41.5)
Married	
No	24 (2.2)
Yes	941 (84.3)
Widow/widower	151 (13.5)
Health insurance	
No	28 (2.5)
National Health Insurance	999 (89.5)
Private insurance	89 (8.0)
Income/month (IDR)	
<2,500,000	650 (58.2)
2,500,000–5,000,000	369 (33.1)
>5,000,000	97 (8.7)

*Note*. FBG = fasting blood glucose; DOQ = Diabetes Obstacles Questionnaire; DQoL = Diabetes Quality of Life; DM = diabetes mellitus; IDR = Indonesian Rupiah.

The S-CVI/ave for all DOQ-30-BI items was .98, with item 8 having the lowest average with a mean (*SD*) score of 3.61 (1.39) and item 28 having the highest average with a mean (*SD*) score of 4.36 (0.95). For all DOQ-30-BI items, skewness scores ranged from −1.465 to −0.190 and kurtosis scores ranged from −1.039 to 1.465.

### Validity of the DOQ-30-BI

#### EFA-assessed Construct Validity

The factor analysis results for all DOQ-30-BI items are shown in Table 2. The analytical results based on fixed factors indicate nine factors. The principal axis factoring with direct oblimin rotation showed a KMO test value of .915, a significant result for Bartlett’s test of sphericity (*p*<.001), and a factor loading value >.49 for all items. These nine factors were: (1) self-monitoring with four items; (2) relationships with medical professionals with four items; (3) knowledge of the disease with five items; (4) support from others with three items; (5) exercise with four items; (6) medication with two items; (7) lifestyle changes with four items; (8) insulin use with two items; and (9) uncertainty about consultation with two items. The loading factors on lifestyle changes and insulin use had negative values, showing the relationship between the observed variables and latent factors to be inversely proportional. A negative loading factor shows the direction of a negative relationship between the variable indicator and its latent construct. Moreover, the higher the variable indicator value, the lower the latent construct value, and vice versa. Although items with negative factor loadings are normally deleted, in this study, considering the significant influence of these items on the QoL of PWDM, these items were retained. Items with negative factor loadings have been retained in prior studies for similar reasons (Doherty et al., 2020).

**Table 2:** Exploratory Factor Analysis of the DOQ-30 Bahasa Indonesia (*N* = 558)

Domain/Item No.	Item	Factor Loading ^a^
Factor 1: Self-monitoring		
22	I find it too uncomfortable to self-monitor	.769
21	Self-monitoring makes me feel frustrated	.736
23	I find it especially hard to test when I am busy	.735
24	Self-monitoring makes me fearful of a high reading	.667
Factor 2: Relationships with Medical Professionals		
2	I have not been told what to expect from my treatment	.919
1	I am not assisted in setting realistic targets for changing my lifestyle	.746
3	The good and bad aspects of each choice have not been discussed with me	.524
4	Treatment alternatives are not explained to me	.505
Factor 3: Knowledge of the Disease		
10	I do not know as much as I need to know to manage my diabetes	.843
11	I do not know as much as I need to know about the consequences of having diabetes	.841
12	I do not know enough about the treatment for diabetes	.695
9	I have difficulty understanding the information from literature	.633
8	I would manage my diabetes much better if I had encouragement socially	.517
Factor 4: Support from Others		
6	I feel I get little support from my friends	.869
5	I feel I get little support from my family	.838
7	I feel very alone with my diabetes	.503
Factor 5: Exercise		
18	I lack the motivation to exercise	.876
19	I am unable to fit exercise into my lifestyle	.861
17	I have not found an exercise I enjoy	.634
20	I am unable to afford the cost of exercising on a regular basis	.494
Factor 6: Medication		
27	I do not feel I am being prescribed a medication dose that is right for me	.872
28	I do not feel I am being prescribed medication that is right for me	.742
Factor 7: Lifestyle Changes		
13	My diabetes has placed a strain on my personal relationships	−.714
14	Changes in my diet have put a strain on my family	−.706
16	My diabetic diet spoils my social life	−.691
15	I feel resentful that I am obliged to change my eating habits	−.635
Factor 8: Insulin use		
30	Using insulin means my diabetes is getting worse	−.818
29	Using insulin makes life too complicated	−.814
Factor 9: Uncertainty about consultation		
26	The way I was told that I had diabetes made me feel afraid	.662
25	I feel a sense of helpless when consulting with nurses	.586

*Note.* DOQ = Diabetes Obstacles Questionnaire.

^a^ Kaiser–Mayer–Olkin= .915; Bartlett’s test of sphericity: degrees of freedom = 435, *p*<.001.

The results of CR and AVE analyses are presented in Table 3. The CR values for individual DOQ-30-BI factors sequentially from the first to the ninth were .818, .778, .837, .791, .816, .790, .781, .799, and .561, while the AVE values for individual sequential factors were .530, .483, .514, .570, .539, .655, .472, .666, and .391. Furthermore, Pearson correlations between factors were calculated to ensure all of the coefficients were correlated (*p*<.01), with *r* values ranging from .231 to .645.

**Table 3:** Reliability Analysis and Convergent Validity of the DOQ-30 Bahasa Indonesia

Domain and Scale Item	Construct Validity (*n*=558)		AVE	CR	Test-Retest Reliability (*n*=100)	
	ITC	Cronbach’s α			ICC	95% CI
All Items		.930			.972	[0.888, 0.988]
Factor 1: Self-monitoring		.848	.530	.818	.962	[0.936, 0.976]
22	.726					
21	.703					
23	.667					
24	.648					
Factor 2: Relationships with Medical Professionals		.760	.483	.778	.929	[0.878, 0.957]
2	.695					
1	.606					
3	.484					
4	.478					
Factor 3: Knowledge of the Disease		.817	.514	.837	.973	[0.960, 0.982]
10	.736					
11	.722					
12	.621					
9	.556					
8	.471					
Factor 4: Support from Others		.769	.570	.791	.911	[0.834, 0.948]
6	.741					
5	.644					
7	.451					
Factor 5: Exercise		.811	.539	.816	.973	[0.953, 0.983]
18	.747					
19	.753					
17	.578					
20	.450					
Factor 6: Medication		.805	.655	.790	.910	[0.857, 0.942]
27	.673					
28	.673					
Factor 7: Lifestyle Changes		.810	.472	.781	.968	[0.933, 0.982]
13	.620					
14	.657					
16	.628					
15	.617					
Factor 8: Insulin use		.828	.666	.799	.932	[0.890, 0.957]
30	.708					
29	.708					
Factor 9: Uncertainty about consultation		.730	.391	.561	.921	[0.878, 0.948]
26	.577					
25	.577					

*Note.* AVE = average variance extracted; CI = confidence interval; CR = composite reliability; DOQ = Diabetes Obstacles Questionnaire; ITC = item total correlation; ICC = interclass correlation coefficient.

#### CFA-assessed Construct Validity

**Figure 1:**
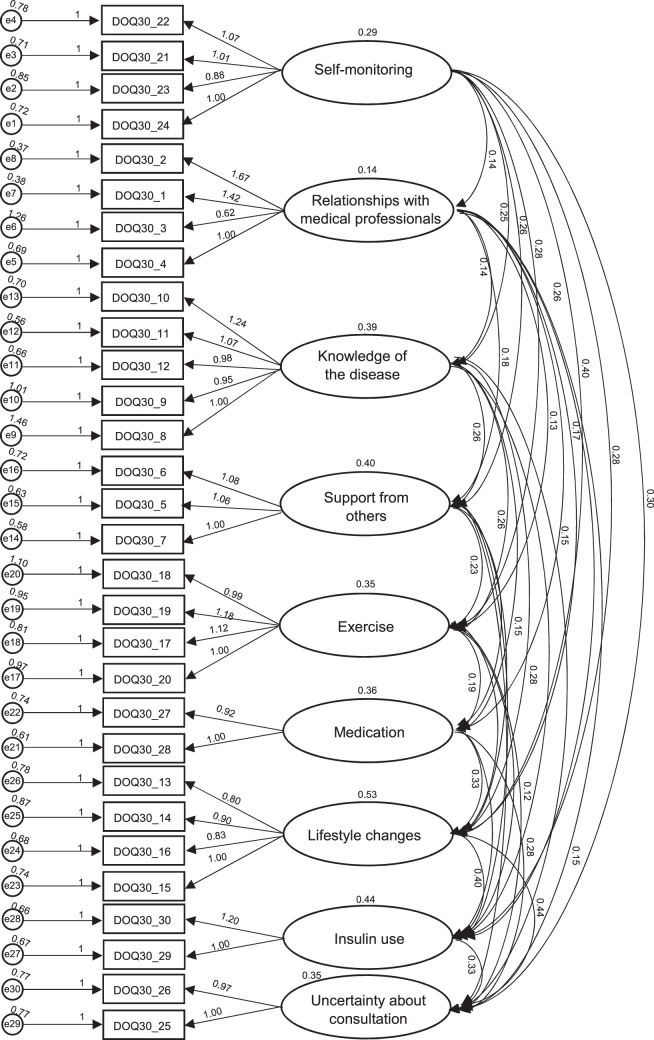
Goodness of Fit of the DOQ-30 Bahasa Indonesia Structural Model

The CFA with nine factors was carried out, with results of χ^2^ = 1038.903, *df* = 369, χ^2^/*df* = 2.815, *p*<.001, GFI = .887, AGFI = .857, IFI = .856, TLI = .827, CFI = .853, RMSEA = .057, and SRMR = .067, indicating the goodness of fit of the model structure. The goodness of fit for the DOQ-30-BI structural model is presented in Figure 1.

#### Convergent Validity

Convergent validity tests for each of the nine factors and all DOQ-30-BI items were conducted in comparison with the DQoL Bahasa Indonesia questionnaire. Overall, DOQ-30-BI results showed a positive and significant relationship with each factor (*r* values range: .482–.617 and *p*<.01). The DQoL questionnaire was shown to relate significantly with the overall DOQ-30-BI (*r* = .711 and *p*<.01) as well as to each of the nine factors (*r* values range: .297–.460 and *p*<.01). Analyses of each DQoL factor found significant relationships between satisfaction (*r* = .386 and *p*<.01), impact (*r* = .658 and *p*<.01), social/vocational (*r* = .661 and *p*<.01), and worry (*r* = .623 and *p*<.01) with the overall DOQ-30-BI. Similarly, significant relationships were identified between each DQoL factor and the nine DOQ-30-BI factors (*r* values range: .060–.447 and *p*<.01, with only satisfaction having six factors *p*<.05).

### Reliability of the DOQ-30-BI

The results for the DOQ-30-BI reliability test are shown in Table 3. The Cronbach’s alpha value was .930 for the overall scale and .848, .760, .817, .769, .811, .805, .810, .828, and .730 for each factor (from 1 to 9). Furthermore, test-retest reliability showed excellent results for all factors, with ICC values ranging from .910 to .968.

## Discussion

To the best of the authors’ knowledge, the DOQ-30 has not previously been translated into other languages, and this was the first study designed to develop and validate the psychometric properties of a Bahasa Indonesia version of the DOQ-30. The results demonstrated the acceptable validity and reliability of test results, which aligns with the original study showing the DOQ-30 to have good internal reliability and external and construct validity based on EFA results (Pilv et al., 2016). The significant importance is that previous studies used EFA only, while this study also conducted CFA and test-retest reliability.

EFA was used to extract factors from the 30 items of the DOQ-30, with the finding that the DOQ-30-BI comprises nine factors with several (two to five) items per factor, which is similar to the original version (Pilv et al., 2016). However, differences between the two may be observed in the order of the factors, with the number of items for each factor previously two to four, and one item was moved to other factors. The item “I would manage my diabetes much better if I had encouragement socially” under the “Support from Friends and Family” factor in the original version was relocated to the “Knowledge of the Disease” factor in this study. The original DOQ-30 study was conducted across six countries in Europe, including Estonia, France, Serbia, Slovenia, Turkey, and Belgium, which, apart from Turkey and Serbia, are all categorized as developed, high-income status countries (Paprotny, 2021). Indonesia, the focus of this study, is a developing country. Even though Serbia and Turkey are developing countries with an upper-middle income status similar to Indonesia, these two countries differ significantly from Indonesia in terms of geographic location, culture, and general level of education. Thus, shifting one item from the “Friends and Family Support” factor to the “Knowledge about the Disease” factor is likely attributable to these differences.

Although prior studies using the DOQ-30 did not report data on participant level of education, average national income influences the educational opportunities available to the population (Ilie et al., 2021). The largest educational level category in this study was high school, followed by elementary school, indicating the participants had a relatively low level of formal education. Level of education influences acquired knowledge, with higher levels associated with more knowledge (Shiferaw et al., 2020). The educational level of the participants in this study may explain their average low level of knowledge, but further studies need to be conducted to confirm. Previous studies have reported a moderate knowledge level among patients with DM in Indonesia (Nabila et al., 2022), which may be expected to result in the low self-management efficacy observed. Thus, due to the low levels of knowledge, PWDM are highly dependent on the social environment (Ligita et al., 2019). Relocating items to other factors has also been done in previous psychometric studies on other topics (Yuliana et al., 2022; Zhang et al., 2017).

The loadings of the nine factors in this study (.494–.919) were higher than those in the original study (.484–.818). Higher item loading onto its respective factors indicates good convergent validity (Cheung et al., 2024). Another construct validity using the CFA strengthened the EFA results, with the resulting model fit indicating the DOQ-30-BI generates valid results. Developers of the original DOQ-30 did not carry out CFA testing, which was a strong reason for us to proceed with CFA testing. As CFA testing has not been conducted by other researchers on the DOQ-30, the results of this study cannot be compared with prior CFA findings. In terms of RMSEA and SRMS values, the CFA with nine factors returned an RMSEA of .057 and SRMR of .067. These results indicate the goodness of fit of the model structure for the DOQ-30-BI.

The results of the reliability tests for each factor and for the total scale revealed good consistency, as evidenced by an overall Cronbach’s alpha coefficient of ≥.70 (range: .730–.848). These results differ slightly from the original version of the DOQ-30, which reported a Cronbach’s alpha value of .52 for the “Uncertainty about consultation” factor and values ≥.75 for all other factors. The original version also did not display Cronbach’s alpha values for the total scale. Also, the ICC values for individual factors in the test-retest assessment in this study were all >.90 with a range of .90–.973, which is high, proving the DOQ-30-BI to be a stable instrument for assessing QoL in people with T2DM. Because the original DOQ-30 study did not conduct a test-retest assessment, comparisons cannot be made. The test-retest approach taken in this study demonstrates that the obtained results are consistent and stable, which further strengthens test reliability.

In addition, the relationship between the DOQ-30-BI and the Bahasa Indonesia version of the DQoL was also examined. The DQoL is another widely used instrument for measuring QoL in patients with DM, and is valid for use on patients with either T1DM or T2DM (Oluchi et al., 2021). When tested using the total item score or per factor, the results showed a positive relationship between the two instruments. The results showed the DOQ-30-BI to be as effective as the DQoL for measuring QoL in patients with DM. The fewer items on the DOQ-30-BI suggest it to be a superior choice, especially for those with T2DM. Items on the DOQ-30-BI also measure all significant QoL aspects related to people with T2DM. While the DQoL primarily assesses the impact of diabetes on an individual’s general well-being, treatment satisfaction, and disease-related worries, the DOQ-30-BI is specifically designed to identify and evaluate perceived *obstacles* or *barriers* that individuals with type 2 diabetes (T2DM) face in managing their condition. These obstacles span various aspects of quality of life (QoL), including emotional, social, and treatment-related domains.

The DOQ-30-BI is a valid and reliable instrument for measuring the barriers that people with T2DM experience when complying with prescribed treatment regimens. The DOQ-30-BI contains comprehensive statement items to provide a more specific picture of patient barriers regarding self-monitoring, relationships with medical professionals, knowledge of the disease, support from friends and family, exercise, medication, lifestyle changes, insulin use, and uncertainty about consultation. This questionnaire takes roughly 15–20 minutes to complete, making it optimized for clinical settings, where limited time is available. The DOQ-30-BI subscales may be used in concert or separately based on patient needs and time available. By detecting obstacles and problems early in people with T2DM, clinicians can carry out early therapy and management to prevent the onset/progression of severe conditions.

Although the DOQ-30-BI is a useful measure of QoL in Indonesians with T2DM, minor limitations should be considered. First, data collection was carried out in only 15 of Indonesia’s 38 provinces. Thus, the generalizability of the results to all people with DM in Indonesia may be limited. Further studies should expand the data collection area and, if possible, cover all provinces in the country. Second, measurements using self-reported questionnaires may introduce recollection and other biases. Counterbalancing these limitations, the advantages of this study include the large number of respondents, which increases the stability and accuracy of validity and reliability estimates. Large sample sizes allow for diversity within a population to be better represented. We also used several approaches to better explain the results when carrying out the cross-cultural adaptation process. Future studies may also examine discriminant validity to further ensure the DOQ-30-BI does not measure factors unrelated to QoL.

### Conclusions

The adequate validity and reliability test results of this study indicate that the DOQ-30-BI has good psychometric properties and accurately measures QoL in the target population. This scale includes 30 items with nine factors: self-monitoring, relationships with medical professionals, knowledge of the disease, support from others, exercise, medication, lifestyle changes, uncertainty about consultation, and insulin use. The DOQ-30-BI is suitable for measuring QoL in Indonesians with T2DM.
